# Intraoperative lung ultrasound improves subcentimetric pulmonary nodule localization during VATS: Results of a retrospective analysis

**DOI:** 10.1111/1759-7714.15027

**Published:** 2023-07-20

**Authors:** Claudio Gambardella, Gaetana Messina, Davide Gerardo Pica, Mary Bove, Francesca Capasso, Rosa Mirra, Giovanni Natale, Francesco Panini D'Alba, Alessia Caputo, Beatrice Leonardi, Maria Antonietta Puca, Noemi Maria Giorgiano, Mario Pirozzi, Stefano Farese, Alessia Zotta, Francesco Miele, Giovanni Vicidomini, Ludovico Docimo, Alfonso Fiorelli, Fortunato Ciardiello, Morena Fasano

**Affiliations:** ^1^ Division of General, Oncological, Mini‐invasive and Obesity Surgery University of Study of Campania “Luigi Vanvitelli” Naples Italy; ^2^ Thoracic Surgery Unit Università degli Studi della Campania “Luigi Vanvitelli” Naples Italy; ^3^ Oncology, Department of Precision Medicine Università della Campania “L. Vanvitelli” Naples Italy; ^4^ General Surgery Unit Università degli Studi della Campania “Luigi Vanvitelli” Naples Italy

**Keywords:** intraoperative lung ultrasound, lung nodules, thoracic surgery, VATS

## Abstract

**Background:**

Video‐assisted thoracoscopic surgery (VATS) resection of deep‐seated lung nodules smaller than 1 cm is extremely challenging. Several methods have been proposed to overcome this limitation but with not neglectable complications. Intraoperative lung ultrasound (ILU) is the latest minimally invasive proposed technique. The aim of the current study was to analyze the accuracy and efficacy of ILU associated with VATS to visualize solitary and deep‐seated pulmonary nodules smaller than 1 cm.

**Methods:**

Patients with subcentimetric solitary and deep‐seated pulmonary nodules were included in this retrospective study from November 2020 to December 2022. Patients who received VATS aided with ILU were considered as group A and patients who received conventional VATS as group B (control group). The rate of nodule identification and the time for localization with VATS alone and with VATS aided with ILU in each group were analyzed.

**Results:**

A total of 43 patients received VATS aided with ILU (group A) and 31 patients received conventional VATS (group B). Mean operative time was lower in group A (*p* < 0.05). In group A all the nodules were correctly identified, while in group B in one case the localization failed. The time to identify the lesion was lower in group A (7.1 ± 2.2 vs. 13.8 ± 4.6; *p* < 0.05). During hospitalization three patients (6.5%; *p* < 0.05) in group B presented air leaks that were conservatively managed.

**Conclusion:**

Intracavitary VATS‐US is a reliable, feasible, real‐time and effective method of localization of parenchymal lung nodules during selected wedge resection procedures.

## Graphical abstract

The use of intraoperative ultrasound facilitates the identification of subcentimetric pulmonary nodules, improving performance in diagnostic and therapeutic terms.

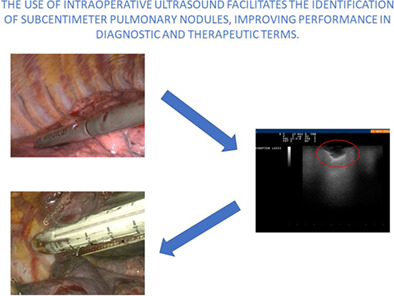

## INTRODUCTION

The incidence of incidental diagnosis of pulmonary nodules has dramatically increased over the last few years.^1^ With the widespread use of high‐resolution computed tomography (HRCT), in fact, a large number of pulmonary nodules are detected annually, but only those with features of malignancy undergo surgical treatment. Surgery with radical intent is the cornerstone of early stage non‐small cell lung cancer (NSCLC) therapy.^1^ Anatomical lobectomy followed by sampling or dissection of mediastinal lymph nodes, is considered the present gold standard of treatment. Conversely, limited resections are reserved for patients with poor performance status.^2^

Khereba et al.^3^ localized 43 of 46 pulmonary nodules by ILU with a 93% success rate^3^, ^4^ Nowadays, minimally invasive thoracic surgery, including robot‐assisted thoracic surgery (RATS) and video‐assisted thoracoscopic surgery (VATS), is the preferred surgical technique for patients with pulmonary nodules due to its advantages in rapid recovery and minimal invasiveness. VATS lobectomy, as suggested in current studies, is a suitable treatment for patients with early stage NSCLC in terms of safety, local control of cancer and survival.^3^, ^5^

The advantages of VATS are the reduction of functional impairment and reduction of postoperative pain, and therefore of general complications, mortality and morbidity.^6^, ^7^

Successful VATS identification of pulmonary nodules depends on their intraoperative research by direct visualization or palpation. The nodules, determined by screening computed tomography (CT) and requiring resection, are nowadays smaller and smaller^8^ and characterization of nodules smaller than 1 cm can be extremely challenging.^9^ Also, solitary and deep‐seated pulmonary nodules are difficult to palpate or identify during VATS.^10^ Methods used to identify the location of pulmonary nodules include bronchoscopic marker placement, CT‐guided percutaneous marker placement, three‐dimensional (3D) printing, intraoperative ultrasound (US), intraoperative molecular imaging (IMI) and artificial intelligence (AI)‐assisted identification.

One of the most promising techniques to achieve the latter target is the adoption of the intraoperative lung ultrasound (ILU) performed during VATS.

The aim of the current study was to analyze the accuracy and efficacy of use of ILU associated with VATS to provide a simple and safe real‐time methodology to visualize solitary and deep‐seated pulmonary nodules smaller than 1 cm compared to VATS alone.

## METHODS

### Study design

This was a retrospective single‐center observational study whose primary aim was to confirm the validity of ILU as a safe and effective localization method to visualize nodules smaller than 1 cm during VATS. The study is reported according to the STROBE statement for cohort studies^11^ and led in compliance with the principles of the Declaration of Helsinki. Written informed consent was obtained from all participants during preoperative communication and the protocol was approved by the Ethics Committee of the University of Luigi Vanvitelli of Naples (32 655/2021). Written informed consent was obtained from all patients.

### Study setting and study population

Patients with solitary and deep‐seated pulmonary nodules less than 1 cm were included in our study from November 2020 to December 2022 at the Thoracic Surgery Department of the Vanvitelli University of Naples.

The inclusion criteria were: a single without characterization deep‐seated pulmonary lesion <1 cm indicated for VATS, age > 18 years, no history of previous thoracic malignant disease, no history of preoperative pulmonary biopsy and no contraindications for surgery. The exclusion criteria were: recent myocardial infarction or unstable angina, severe neurological problems, a prolonged prothrombin time (PT‐INR) >1.5 or a platelet count <30 000, impossibility to tolerate single lung ventilation and pregnancy, emphysema and patients with nodules close to the hilar zone.

All subjects were preoperatively assessed during a specialized thoracic surgery evaluation.

All patients underwent preoperative HRCT, contrast‐enhanced CT, and ^18^F‐fluorodeoxyglucose positron‐emission tomography/computed tomography (^18^F‐FDG PET/CT) to record the localization and size of the lesions. All surgeries were performed by experienced thoracic surgeons who had performed over 300 thoracic oncology procedures using VATS and were experienced in pulmonary ultrasound. Clinical data were collected from a prospectively maintained electronic database. Patients who received VATS aided with ILU were considered as group A and patients who received conventional VATS were in group B and considered the control group. All patients considered in the analysis presented with without characterization, PET‐enhanced, subcentimetric deep‐seated pulmonary nodules; therefore an extemporaneous intraoperative examination was necessary in any case to address the surgery.

### Conventional VATS

Patients were placed in a lateral decubitus position, general anesthesia was induced, and double‐lumen endotracheal intubation with contralateral single lung ventilation was performed; patients underwent ultrasound‐guided fascial plane blocks of the chest wall using long‐lasting local anesthesia in order to reduce postoperative pain. Lung specimens were not ventilated but rather semi‐inflated and inflated.

The VATS approach employed was the anterior triportal approach according to Hansen et al. which consists of two 1–1.5 cm lower access incisions, located in the seventh or eighth intercostal space, in the posterior and anterior axillary lines, respectively, for two thoracoscopic ports and a 4–5 cm port incision, placed in the fourth intercostal space, in the anterior axillary line for a utility incision.^12^

After careful exploration of the cavity and identification of the nodule, the surface of the nodules was cauterized with a cautery stick, then a wedge resection with a 2 cm margin was performed; the specimen was sent to the department of pathology to confirm the accuracy of excision.

### VATS associated with ILU

Patients were placed in a lateral decubitus position, general anesthesia was induced, and double‐lumen endotracheal intubation with contralateral single lung ventilation was performed; patients underwent ultrasound‐guided fascial plane blocks of the chest wall using long‐lasting local anesthesia in order to reduce postoperative pain. Lung specimens were not ventilated but rather semi‐inflated and inflated.

The VATS approach employed was the anterior triportal approach according to Hansen et al.,^12^ which consists of two 1–1.5 cm lower access incisions, located in the seventh or eighth intercostal space, in the posterior and anterior axillary lines, respectively, for two thoracoscopic ports and a 4–5 cm port incision, placed in the fourth intercostal space, in the anterior axillary line for a utility incision.^13^

**FIGURE 1:**
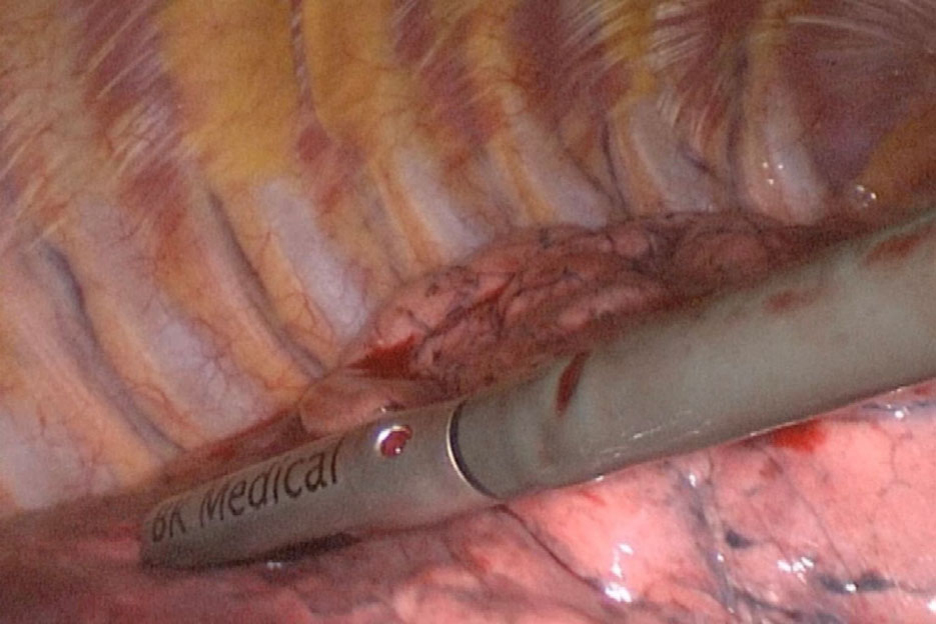
The BK 5000 ultrasound (US) processor was used for the localization of small lung nodules. A sterile intracavitary laparoscope probe 38 cm in length, 10‐mm in diameter and a flexible tip, equipped with a convex array transducer with frequencies ranging from 4 to 12 MHz, was introduced through one of the video‐assisted thoracoscopic surgery (VATS) ports.

A BK 5000 ultrasound (US) processor was used for the localization of small lung nodules. A sterile intracavitary laparoscope probe 38 cm in length, 10‐mm in diameter and a flexible tip, equipped with a convex array transducer with frequencies ranging from 4 to 12 MHz, were introduced through one of the VATS ports (Figure 1). A setting for superficial tissue with tissue harmonics, electronic focusing at the planar interface level and time gain <50%, were used. The probe was inserted into the chest through the operating hole, and the mediastinal, costal and diaphragmatic surfaces of the lung were explored for subcentimetric nodules. These can only be visualized when the lung is completely deflated. The surgeon, applying light pressure on the lung surface with the ultrasound probe, reducing residual air, localizes deeper nodules if possible. During the examination, the probe was perpendicular to the pulmonary surface and warm sterile saline was used to improve surface contact.

VATS‐US was adopted to identify size, localization and US pattern of the lesions of interest.

In cases where pulmonary nodules were found their ultrasound characteristics were recorded.

**FIGURE 2:**
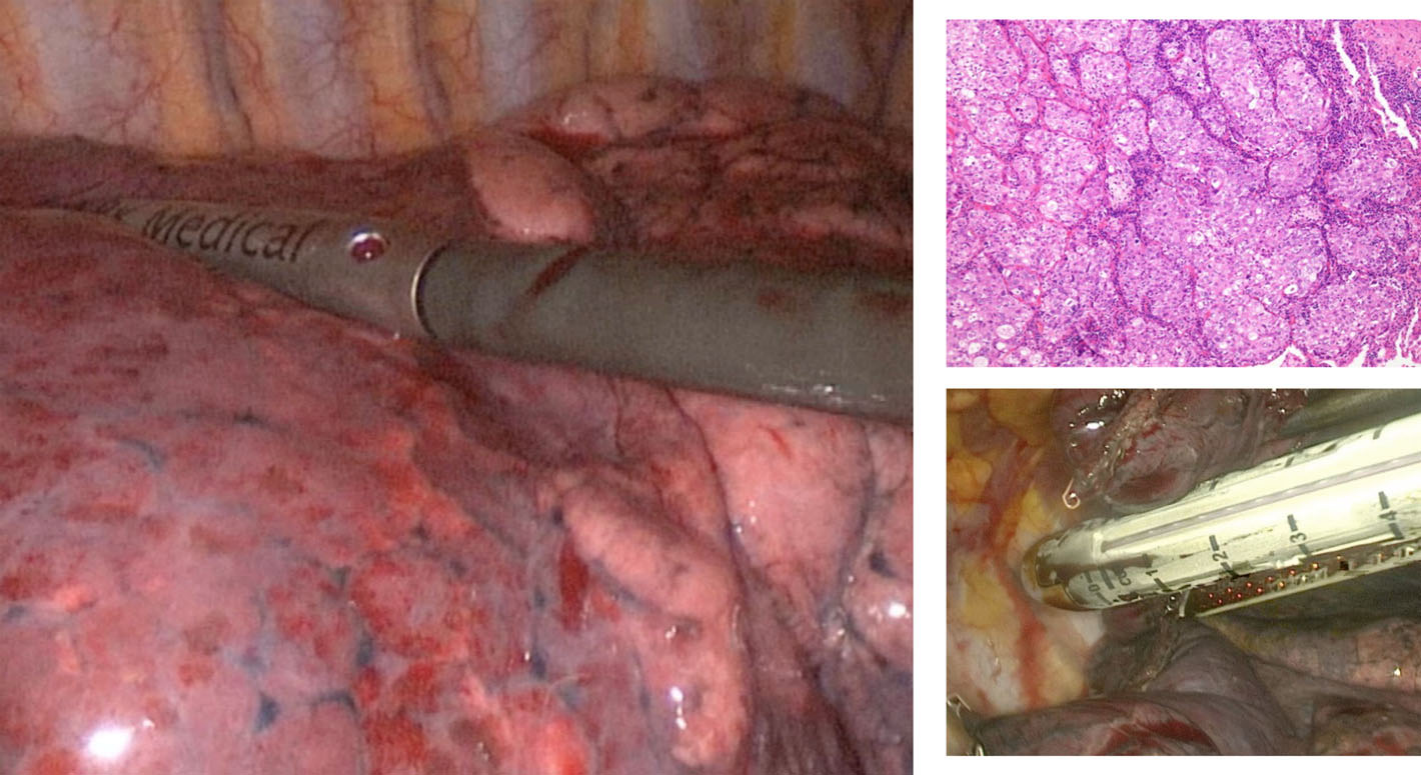
An ultrasound (US) probe was inserted by an expert ultrasound surgeon into the chest through the operating hole and light pressure was applied on the lung surface for localization of lung nodules.

Subsequently, the surface of the nodules was cauterized with a cautery stick, then a wedge resection with a 2 cm margin was performed; the specimen was sent to the department of pathology to confirm the accuracy of excision (Figure 2).

### Ultrasound parameters of pulmonary nodules

**FIGURE 3:**
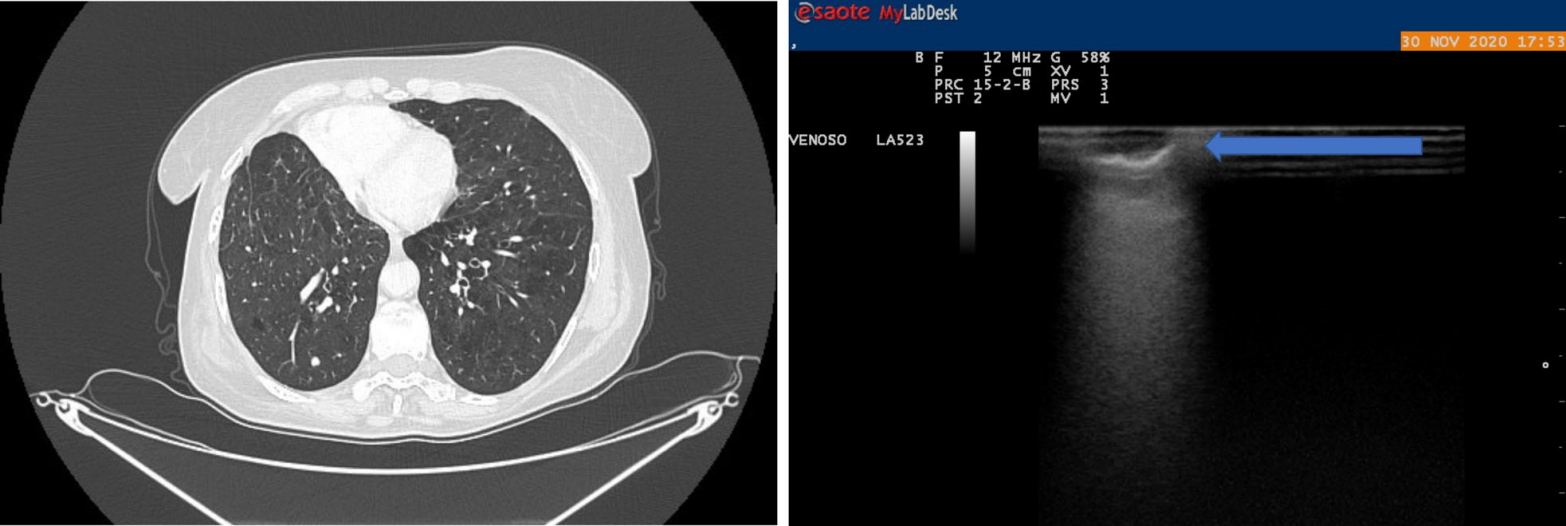
Irregular shaped lung nodule that was hypoecoic on ultrasound. Hyperecoic striae are present.

**FIGURE 4:**
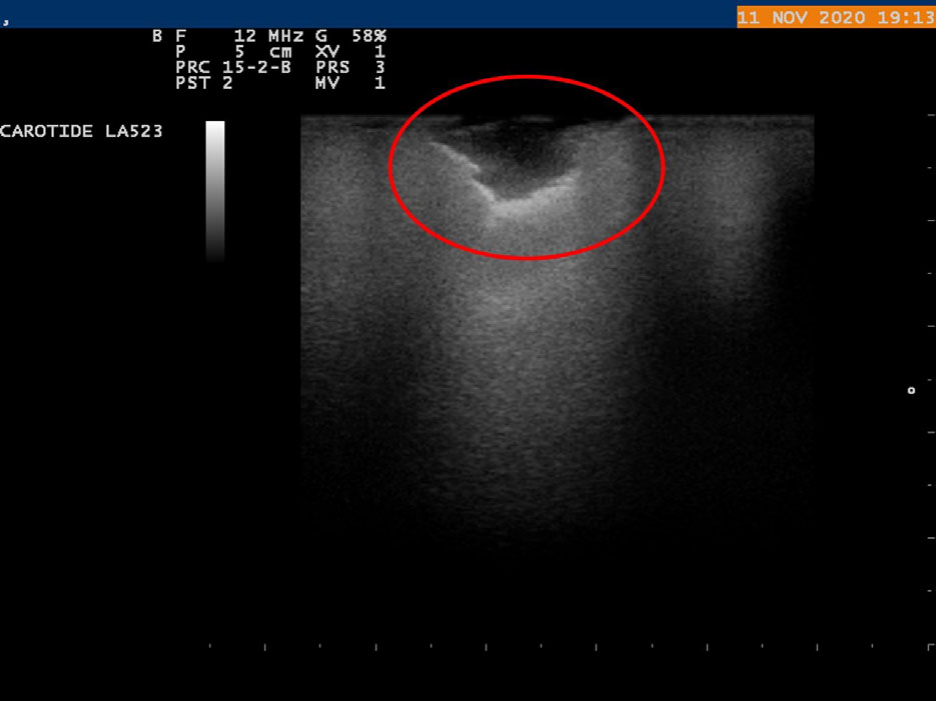
Irregular shaped lung nodule that was hypoecoic on ultrasound. Hyperecoic striae are present.

**FIGURE 5:**
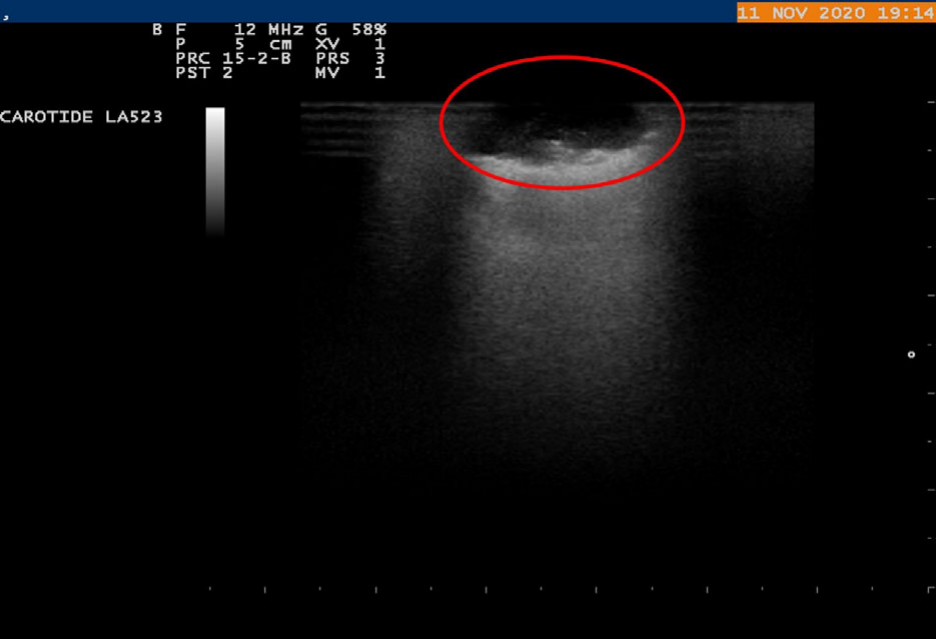
Irregular shaped lung nodule that was hypoecoic on ultrasound. Hyperecoic striae are present.

With the aid of the ILU, the margins of the nodules were assessed and classified as “well defined” or “jagged”, according to their shape as “regular” or “irregular” and according to their echogenicity, as hypoechoic or hyperechoic (Figures 2 and 3). The presence or absence of inner hyperechoic striae and/or spots was assessed. “Size” was defined as the mean between the maximum and minimum diameter of the nodule^14^ (Figures 3, 4, 5).

### Outcome measures

Mean overall operative time was evaluated in minutes. Mean time for the identification of the nodules was also assessed in minutes. The presence of any perioperative complications (i.e., air leak or hemorrhage) was recorded and reported during hospitalization. The rate of identification of the nodules was expressed as the percentage nodules identifiable with the VATS alone and with the VATS aided with ILU in each group.

### Study outcomes

The primary outcome of the current study was the analysis of the identification rate and time of subcentimetric solitary and deep‐seated pulmonary nodules in patients who had undergone VATS alone and VATS aided with ILU.

The secondary outcome was the evaluation of perioperative complications and hospitalization in patients who had undergone VATS alone and VATS aided with ILU.

### Statistical analysis

Statistical analysis was performed using Excel 2011 (Microsoft) and via the Graph Pad Prism 9 program according to recently published guidelines.^15^ Categorical data are reported as raw numbers with percentages in parenthesis. Continuous data are reported as means ± standard deviation or as medians with range in parenthesis, according to the distribution. The differences between results were analyzed using an unpaired *t*‐test if they were summarized as means, the Mann Whitney U test if they were summarized as medians, or the Fisher's exact test if they were reported as percentages. For dichotomous variables (presence or absence of a condition), counts were made in the subgroups without performing probabilistic tests. The variables that were found to be significantly equal in the comparison between subgroups (that is, with the same mean or the same percentage composition in the subgroups) were considered neutral for the purpose of determining the final outcome. A *p*‐value less than 0.05 was considered statistically significant.

## RESULTS

### Study population

From November 2020 to December 2022 of 83 patients referred for isolated solitary and deep‐seated pulmonary nodules smaller than 1 cm, 74 met the inclusion criteria and underwent a pulmonary wedge resection. Forty‐three patients received VATS aided with ILU (group A) and 31 patients received conventional VATS (group B). The demographic and preoperative findings are detailed in Table 1.

**TABLE 1:** Demographic and pathological findings.

Findings	Group A (*n* = 43)	Group B (*n* = 31)	*p*‐value
Age, years*	62.3 ± 7.3	64.7 ± 6.4	0.243
Gender, male/female	28/15 (60.1%/39.9%)	18/13 (61.1%/38.9%)	0.537
BMI	28.3 ± 7.3	27.4 ± 5.3	0.172
Preoperative symptoms			
Cough	8 (18.6%)	4/31 (12.9%)	0.511
Asthenia	3 (6.9%)	2 (6.4%)	0.703
Comorbidities			
Cardiovascular diseases	5 (11.6%)	3 (9.7%)	0.789
Diabetes	11 (25.6%)	8 (25.8%)	0.982
COPD	7 (16.2%)	4 (12.9%)	0.687

*Note*: Values are expressed as means ± standard deviation or as raw numbers with percentages in parenthesis.

Abbreviations: LLL, left lower lobe; LUL, left upper lobe; ML, medium lobe; RLL, right lower lobe; RUL, right lower lobe; VATS, video‐assisted thoracoscopic surgery.

### Primary outcome

Mean operative time was 84 ± 7.3 min in group A and 99 ± 4 min in group B (*p* < 0.05). No significant intraoperative complications occurred. In group A all the nodules were correctly identified (43/43, 100%), while in group B, in one case (30/31, 96.7%), the localization failed and it was necessary to enlarge the incision to about 2 cm, in order to facilitate the insertion of the hand and the localization of the nodule by finger palpation. This event increased the difficulty of the operation.^14^ The time to identify the lesion was lower in group A (7.1 ± 2.2 vs. 13.8 ± 4.6; *p* < 0.05). Moreover, as a result of the difficulty in nodule sampling in three cases in group B, further resection and intraoperative histological examination were performed. The median range of the depth from the pulmonary surface was 3.9 ± 2.2 versus 4.2 ± 1.5 in group A and group B (*p* = 0.679), respectively. After the pathological response, the patients showing squamous carcinoma (27 patients in group A and 19 patients in group B) or adenocarcinoma (9 patients in group A and 6 patients in group B) underwent lobectomy. Conversely, wedge resection was adequate in cases of breast (group A 3 and group B) or colon (group A 4 and group B 3) metastases. The intraoperative and pathological findings are summarized in Table 2.

**TABLE 2:** Intraoperative and pathological findings.

Findings	Group A (*n* = 43)	Group B (*n* = 31)	*p*‐value
Mean operative time, min*	84 ± 7.3	99 ± 4.8	<0.05
Area of lung nodules, *n* (%)			
RUL	14 (32.5%)	11 (35.5%)	0.792
LLL	11 (25.6%)	7 (22.6%)	0.766
ML	7 (16.3%)	4 (12.9%)	0.687
RLL	4 (9.3%)	6 (19.3%)	0.212
LUL	7 (16.3%)	3 (9.7%)	0.412
Nodules diameter, mm*	7.3 ± 1.2	8.4 ± 1.5	0.124
Distance from the visceral pleura, mm*	16.2 ± 2.4	15.5 ± 3.2	0.148
Time to identify the nodule, min*	7.1 ± 2.2	13.8 ± 4.6	<0.05
Lung nodules identification, *n* (%)	43/43 (100%)	30/31 (96.7%)	0.235
Surgical resections, *n* (%) LLL wedge resection RUL wedge resection LUL VATS lobectomy LLL VATS lobectomy RUL VATS lobectomy ML VATS lobectomy RLL VATS lobectomy	3 (6.9%) 2 (4.7%) 7 (16.2%) 11 (25.6%) 10 (23.4%) 8 (18.6%) 2 (4.6%)	3 (9.7%) 2 (6.4%) 5 (16.1%) 7 (22.6%) 6 (19.4%) 5 (16.1%) 3 (9.7%)	0.674 0.735 0.986 0.766 0.687 0.782 0.395
Intraoperative complications, *n* (%)	0	0	–
Histology, *n* (%)			
Squamous carcinoma	27 (62.8%)	19 (61.3%)	0.895
Adenocarcinoma	9 (20.9%)	6 (19.3%)	0.867
Colon metastasis	4 (9.4%)	3 (9.7%)	0.956
Breast metastasis	–	3 (9.7%)	–
Prostate metastasis	3 (6.9%)	–	–

*Note*: Values are expressed as means ± standard deviation or as raw numbers with percentages in parenthesis.

Abbreviations: LLL, left lower lobe; LUL, left upper lobe; m, minutes; ML, medium lobe; RLL, right lower lobe; RUL, right lower lobe; VATS, video‐assisted thoracoscopic surgery.

### Secondary outcome

The mean hospitalization was 3.1 ± 5.3 days in group A and 3.5 ± 7.1 days group B (*p* = 0.287; unpaired *t*‐test). During hospitalization three patients (6.5%; *p* < 0.05) in group B had air leaks that were conservatively managed maintaining in site the chest drainage for 10 days (Table 3). In the other patients in both groups the chest tube was removed on the third postoperative day.

**TABLE 3:** Postoperative findings.

Findings	Group A (*n* = 43)	Group B (*n* = 31)	*p*‐value
Hospitalization, days*	3.1 ± 5.3	3.5 ± 7.1	0.287
Perioperative complications, *n* (%)			
Air leak	0	3 (6.5%)	0.037
Hemorrhage	0	0	–
Empyema	0	0	–
Pneumonia	0	0	–

*Note*: Values are expressed as means ± standard deviation or as raw numbers with percentages in parenthesis.

## DISCUSSION

The introduction of minimally invasive thoracic surgery and consequently the intraoperative localization of subcentimetric pulmonary nodules has become very important in early lung cancer, and VATS has been adopted as an important tool in the treatment of this devastating disease through minimally invasive surgery. VATS is especially useful in patients with clinically debilitated conditions or with marginal pulmonary reserves. In fact, thoracoscopy is associated with less postoperative pain and morbidity and earlier recovery than open thoracotomy.^12^, ^13^, ^14^, ^15^, ^16^

Intraoperative ultrasound during VATS origins from laparoscopic surgery; intraoperative high‐frequency ultrasound probes are used to detect small and deep lesions with a size of 10 mm or less in the lung or mediastinum and to stage lung lesions. Therefore, the use of intraoperative ultrasound was doubted for localization purposes in the lung because the air in the parenchyma often inhibits proper ultrasound examination; pulmonary collapse is mandatory for nodule localization, nevertheless failure to detect a lesion may require conversion to thoracotomy. Failure of localization of pulmonary nodules less than 1 cm in VATS has previously been reported in up to 16.1% of cases and, in these studies, minimally invasive surgery was converted to open thoracotomy in up to 54% of cases.^17^, ^18^

Different preoperative techniques have the purpose of marking the nodule favoring its localization, such as microcoiling, indocyanine tattooing,^19^ radiolabeling and hooking.^20^ Although these methods have a success rate up to 100% and open thoracotomy may be avoided in over 50% of cases, complications such as pneumothorax, air embolism, intraparenchymal and bleeding is about 16.7%.

Moreover, hook wire dislodgment from the pulmonary parenchyma is not a rare event; it requires two different spaces including the computed tomographic facility and the operating room.

Successes are operator‐dependent and require a multidisciplinary approach that often involves a high‐cost.

Advances in imaging technology in ultrasound (US) have resulted in a higher rate of utilization of this procedure.^21^ VATS with a triportal approach has been found to reduce postoperative complications, such as functional impairment, pain and mortality, decreased operation time and intraoperative hemorrhage when compared to open surgery.^22^, ^23^, ^24^ Matsumoto et al.^24^ detected 25 pulmonary nodules using ILU. However, in three cases nodules were neither visible nor palpable.

However, the main limitation of VATS is that palpation of the lung surface is not always possible, in particular in cases of small or deep lesions.^25^ Lung palpation, in fact, could be difficult during VATS due to the limited area that can be reached by the operator's finger and the increased risk of major complications. Therefore, surgeons are searching for additional techniques for their localization.^26^ The benefits of VATS are reduced by missing the localization of target lesion or by spending a lot of time searching for it.^27^, ^28^ According to Hou et al., the palpation method was considered to have failed if it took more than 12 minutes.^27^

The detection of central nodules necessitates complete desufflation of the lung to avoid imaging artifacts; consequently, a systematic evaluation of the pulmonary parenchyma without artifacts due to the high acoustic impedance difference encountered by the ultrasound beam at the interface between the aerated lung parenchyma and the overlying chest wall soft tissues should be performed. Moreover, a strict collaboration with the anesthesiological equipment is essential. Therefore, ILU is a complementing, real‐time and safe method for the localization of small nodules.^29^, ^30^ It is a new technique that necessitates development for its ability to exactly localize invisible or nonpalpable pulmonary nodules in real‐time during VATS,^31^, ^32^ in order to perform more diagnostic biopsies and achieve safe surgical margins.^33^, ^34^

To the best of our knowledge, this is the first study in the literature to analyze the efficacy of ILU during VATS in identifying subcentimetric intraparenchymal nodules that lie at a depth up to 5 cm from the pulmonary surface. In the current study no intraoperative complications occurred in patients who underwent ILU in association with VATS. With the aid of ILU all the nodules were correctly identified (43/43, 100%), while with VATS alone the localization failed in one case (30/31, 96.7%) and it was necessary for the incision to be enlarged by about 2 cm, in order to facilitate insertion of the hand and localization of the nodule by finger palpation making the surgery more challenging. Moreover, the time to identify the lesion was lower in group A (7.1 ± 2.2 vs. 13.8 ± 4.6; *p* < 0.05), guaranteeing important benefits, especially in unfit patients. Therefore, in group A the use of a last generation ultrasound machine allows the study of all the pulmonary parenchyma, the easy identification of several anatomical landmarks and then the easier and quick detection of the subcentimetric centroparenchymal nodule. Moreover, as a result of the difficulty in nodule sampling of three cases in group B, a further resection and further intraoperative histological examination were necessary. The ultrasound localization was necessary in the first phase since all patients considered in the analysis had without characterization, PET‐enhanced, subcentimetric pulmonary nodules; therefore, an extemporaneous intraoperative examination was necessary in any cases to address the surgical procedure. The use of intraoperative ultrasound, considering the ultrasonographic features of the nodules, allows us to easily identify suspicious nodule that should be examined, even if at 5 cm depth, avoiding unnecessary resections and performing a parenchyma sparing sampling. The identification of suspicious nodules in such conditions with palpation alone during conventional VATS, in fact, could be extremely challenging.

Subsequently, after the pathological response, the patients in cases of extemporaneous examination showing squamous carcinoma (27 patients in group A and 19 patients in group B) or adenocarcinoma (9 patients in group A and 6 patients in group B) underwent lobectomy. Conversely, wedge resection was adequate in cases of breast (3 in group A and 3 in group B) or colon (group A 4 and group B 3) metastases.

Mean hospitalization was lower in group A (3.1 ± 5.3 days vs. 3.5 ± 7.1 days), even if not statistically significant (*p* = 0.287). Noteworthy, during hospitalization three patients (6.5%; *p* < 0.05) in group B presented air leaks that were conservatively managed, while the postoperative course in group A was free from complications. However, the study by Kondo et al. demonstrated that ultrasound‐trained surgeons can safely and effectively locate nonpalpable nodules using intraoperative ultrasound.^9^ Other studies have confirmed that the success of intraoperative ultrasound investigation of pulmonary nodules can be really promising (93%–98%).^4^, ^24^, ^35^, ^36^ Imperatori et al., in their comparative series, were able to detect all the pulmonary nodules using ILU (assessment percentage 100%), compared to about 95% by finger palpation. Most of the lung nodules included (96%) were <2 cm in size and the dimension of the seven nodules that escaped detection by digital palpation was statistically lower compared to that of palpable nodules.

Hou et al. also showed that the localization success rate of the subcentimetric pulmonary nodules by ILU was 100%, suggesting that ILU could detect nodules within a certain range of size and depth.^27^, ^37^

Noteworthy, during VATS, US allowed us to explore almost completely the visceral pleura; the intraoperative ultrasound probe, in fact, can reach areas of the lung that cannot be reached by finger palpation alone. Thus, ultrasound also appears to be a compensative procedure in case of the failure of CT‐guided intraoperative marking.

This study had some limitations. First, ILU is an operator‐dependent technique which requires large experience with a variable learning curve. Therefore, it is performed only in tertiary level hospitals. Therefore, the methods are not easily reproducible, and the results are not easily generalizable. Moreover, in patients with emphysema the ultrasound localization of the nodules is more challenging and were not considered in the current study. Finally, other limitations were the limited number of cases reported and the retrospective design of the study.

In conclusion, intracavitary VATS‐US is a reliable, feasible, real‐time and effective method of localization of parenchymal lung nodules during selected wedge resection procedures that in the reported series showed excellent results in terms of intra‐ and postoperative complications and regarding the accuracy and timing of the localization. As with any ultrasound procedure, ILU requires a long training and experience, especially for the problems related to the not correctly collapsed lung.

In the current series, intraoperative ultrasound during VATS proved to be a safe and reliable method for the real‐time identification of pulmonary nodules not evaluated by digital palpation, therefore IUS is extremely useful for subcentimetric nodules which, being centrally located or in any case not being in a subpleural position, they can more easily escape the touch of the surgeon during manual palpation.

## AUTHOR CONTRIBUTIONS

All authors had full access to the data in the study and take responsibility for the integrity of the data and the accuracy of the data analysis. Conceptualization, Claudio Gambardella and Gaetana Messina; Methodology, Davide Gerardo Pica, Giovanni Natale; Investigation, Mary Bove, Fortunato Ciardiello, Rosa Mirra and Beatrice Leonardi; Formal Analysis, Mario Pirozzi and Stefano Farese; Resources, Alessia Zotta and Morena Fasano; Writing ‐ Original Draft, Gaetana Messina, Giovanni Vicidomini; Writing ‐ Review & Editing, Alfonso Fiorelli, Ludovico Docimo, Noemi Maria Giorgiano, Francesco Panini D'Alba; Visualization, Francesca Capasso; Supervision, Alfonso Fiorelli; Funding Acquisition, Francesca Capasso.

## FUNDING INFORMATION

University of Campania L. Vanvitelli.

## CONFLICT OF INTEREST STATEMENT

The authors have no conflicts of interest to declare.

## Data Availability

Authors can confirm that all relevant data are included in the article and/or its supplementary information files.
